# Nursing Staff Perceptions of Risk Outcomes in Delivering Preference-Based Person-Centered Care

**DOI:** 10.1093/geroni/igab046.1039

**Published:** 2021-12-17

**Authors:** Liza Behrens, Marie Boltz, Ann Kolanowski, Mark Sciegaj, Katherine Abbott, Caroline Madrigal, Kimberly Van Haitsma

**Affiliations:** 1 Ross and Carol Nese College of Nursing, Pennsylvania State University, University Park , Pennsylvania, United States; 2 Pennsylvania State University, University Park, Pennsylvania, United States; 3 Penn State, University Park, Pennsylvania, United States; 4 Penn State University, University Park, Pennsylvania, United States; 5 Miami University, Oxford, Ohio, United States; 6 Providence VA Medical Center, Providence VA Medical Center, Rhode Island, United States; 7 The Pennsylvania State University, University Park, Pennsylvania, United States

## Abstract

Effective management of the perceived risks associated with delivering preference-based person-centered care (PBPCC) is historically challenging for nursing home staff. Existing research lacks the granularity needed to guide clinicians who fear negative health and safety outcomes for residents. This study examined direct-care nursing staff perceptions of outcomes associated with delivering PBPCC. Participants (N=27) worked in NHs experiencing 6-12 health citations, were mostly female (85%), and represented diverse ages, race, education, and collective work experience in NHs. Content analysis of verbatim transcripts from 12 focus groups identified an overarching theme of: “person-centered outcomes related to risk engagement”; and sub-themes of: harms to staff (e.g. fear, frustration, guilt); harms to residents (e.g. negative moods and behaviors, physical discomfort); and positive shared outcomes (e.g. building nurse-resident relationships, positive care environment). Implications for risk management that improves quality of care and life outcomes in a post-COVID era will be discussed.

